# Genetic Attributes of *E*. *coli* Isolates from Chlorinated Drinking Water

**DOI:** 10.1371/journal.pone.0169445

**Published:** 2017-01-20

**Authors:** Michaela D. J. Blyton, David M. Gordon

**Affiliations:** Evolution, Ecology and Genetics, Research School of Biology, The Australian National University, Acton, ACT, Australia; Natural Environment Research Council, UNITED KINGDOM

## Abstract

*Escherichia coli*, is intimately associated with both human health and water sanitation. *E*. *coli* isolates from water can either be (i) host associated commensals, indicating recent faecal contamination; (ii) diarrheal pathogens or (iii) extra-intestinal pathogens that pose a direct health risk; or (iv) free-living. In this study we genetically characterised 28 *E*. *coli* isolates obtained from treated drinking water in south eastern Australia to ascertain their likely source. We used full genome sequencing to assign the isolates to their phylogenetic group and multi-locus sequence type. The isolates were also screened in silico for several virulence genes and genes involved in acquired antibiotic resistance. The genetic characteristics of the isolates indicated that four isolates were likely human pathogens. However, these isolates were not detected in sufficient numbers to present a health risk to the public. An additional isolate was a human associated strain. Nine isolates were water associated free-living strains that were unlikely to pose a health risk. Only 14% of the isolates belonged to the host associated phylogenetic group (B2) and only a single isolate had any antibiotic resistance genes. This suggests that the primary source of the drinking water *E*. *coli* isolates may not have been recent human faecal contamination.

## Introduction

The bacterium, *Escherichia coli*, is intimately associated with both human health and water sanitation. It is a ubiquitous commensal of the mammalian gastro-intestinal tract and this attribute combined with its generally poor survival in the external environment, has led to its use as an indicator of recent faecal contamination and therefore of the potential occurrence of enteric pathogens [1]. Some strains of *E*. *coli* are known diarrheal pathogens [2, 3] and others can opportunistically cause extra-intestinal infections, particularly of the urinary tract [4]. These pathogenic strains often possess particular virulence associated genes that make them genetically distinct from commensal strains [3, 5]. However, not all *E*. *coli* strains are host associated. There is growing evidence that some *E*. *coli* strains are free-living in water bodies [6]. These strains can be responsible for elevated cell counts in water ways that are independent of faecal contamination and are unlikely to pose a public health risk. Therefore, *E*. *coli* strains isolated from a water sample may be (i) host associated commensals, indicating recent faecal contamination; (ii) diarrheal pathogens or (iii) extra-intestinal pathogens that pose a direct health risk; or (iv) free-living.

In most developed countries, raw water from large storage dams is treated so that it is safe for human consumption before it is distributed to the public. In Australia, organic matter, sediment and minerals are removed from the water where necessary and it is disinfected using chlorine and/or ultra-violet radiation. The water is tested prior to and post treatment to verify water quality. *E*. *coli* is normally killed by the addition of chlorine, however, in rare cases *E*. *coli* is detected in the treated water samples (1 in 1000 samples) [7]. It is unclear whether these cells have survived the treatment process or whether they represent post treatment contamination. To better understand the risk *E*. *coli* isolated from chlorinated drinking water pose to human health, detailed characterisation of such isolates is required.

Due to the rarity with which *E*. *coli* is isolated from chlorinated drinking water, sufficient sample sizes can seldom be achieved for meaningful analysis of their general characteristics. In this study we had the unique opportunity to genetically characterise a sufficient number *E*. *coli* isolates obtained from treated drinking water in south eastern Australia to ascertain their likely source and the risk to public health. The isolates were characterised using full genome sequencing to assign them to their phylogenetic group and multi-locus sequence type. They were also screened in silico for several virulence genes and genes involved in acquired antibiotic resistance.

## Materials and Methods

### Strains

Twenty-eight *E*. *coli* isolates that were detected in chlorinated drinking water between November 2010 and March 2014 were screened in silico for their genetic attributes. The isolates were provided by three South Eastern Australian water distribution companies and a private laboratory that undertakes water testing on behalf of water authorities. Each isolate represented a solitary colony recovered from a water sample. In some cases multiple *E*. *coli* isolates were detected at different points in the distribution network on the same day, while on other occasions, single *E*. *coli* isolations occurred. The isolates were sent to the Australian National University for genetic analysis.

### Genome Sequencing

For the genome sequencing, DNA extractions were performed by inoculating the isolates into 1 ml of Lysogeny broth and incubating for 19 hours at 37°C. DNA was then extracted from 100 μl of the cell cultures using the Isolate II genomic DNA kit (Bioline) with a 1 hour digestion, according to the manufacturer’s instructions.

Sequencing libraries were prepared from the extracted DNA using the Nextera XT sample preparation kit (Illumina) following the manufacturer’s protocol. Each library was uniquely tagged using the Nextera XT indexing primers (Illumina) and 24 libraries were pooled in a sequencing run. Paired-end sequencing was performed at the Biomolecular Resource Facility, Australian National University, on the Illumina Miseq using the version 2 reagent kit for 250 cycles. The raw sequence read files have been deposited in GenBank and are associated with SRA study SRP089829.

### Bioinformatics

The sequencing reads were processed and assembled (*de novo*) in CLC genomic workbench 7.0 using the default settings (minimum read length = 50, maximum read length = 800, word size = 20, bubble size = 50, minimum contig length = 500). The reads were then mapped back to the contigs using the following parameters: mismatch cost = 2, insertion cost = 3, deletion cost = 3, length fraction = 0.5 and similarity fraction = 0.8. The assembled genomes were then screened in silico for their multilocus sequence types, intestinal virulence and antibiotic resistance gene profiles using a series of online software tools provided by the Centre of Genomic Epidemiology (CGE, http://www.genomicepidemiology.org/). The extra-intestinal virulence gene profile and phylogenetic group membership of each isolate was also determined in silico.

#### Phylogenetic group

Partial sequences for 12 core house-keeping genes were extracted from the full genomes of the isolates using the CGE MLST screening tool described in Larsen et al. [8]. The genes were *adk* (536 bp), *fumC* (469 bp), *icd* (516 bp), *gyrB* (460 bp), *mdh* (452 bp), *pabB* (468 bp), *polB* (450 bp), *purA* (478 bp), *putP* (456 bp), *recA* (510 bp), *trpA* (561 bp) and *trpB* (594 bp). A phylogenetic tree was then constructed from the concatenated sequences (5950 bp) in Mega version 6 [9]. In addition to the 28 tap isolates, 96 representative *E*. *coli* strains [10] were included in the analysis to ensure the different phylogenetic groups could be delimitated in the tree. *Escherichia fergusonii* and *Escherichia albertii* were included as out groups. The tree was inferred by using the Maximum Likelihood method based on the General Time Reversible model [11]. Initial tree(s) for the heuristic search were obtained by applying the Neighbour-Joining method to a matrix of pairwise distances estimated using the Maximum Composite Likelihood (MCL) approach. A discrete Gamma distribution was used to model evolutionary rate differences among sites (6 categories). The rate variation model allowed for some sites to be evolutionarily invariable.

#### MLST

The multi-locus sequence types (MLST) of the isolates were determined using the CGE MLST screening tool described in Larsen et al. [8]. The isolates were screened in silico for the seven gene typing scheme of Wirth et al. [12].

#### Virulence associated genes

The isolates were screened in silico using the CGE VirulenceFinder 1.2 tool for the presence or absence of a set of 76 virulence genes, including genes responsible for verotoxigenesis [13]. This set of loci included genes used to identify probable gastro-intestinal pathogens including typical and atypical entropathogenic (EPEC), enteroinvasive (EIEC), enterotoxigenic (ETEC), enterohemorrhagic (EHEC), and shiga toxin-producing (STEC) *E*. *coli* as defined by Robins-Browne et al. [3]. The identity threshold was set to 85% for a positive match to be identified between a target genome and the reference database. As the gene *gad* is present in all *E*. *coli* strains, this gene was not included in the results or analyses.

The isolates were also screened in silico for an additional panel of 34 extra-intestinal associated virulence genes in CLC genomic workbench 7.0. This panel included five cardinal extra-intestinal associated genes as defined by Johnson et al. [5]. Johnson et al. [5] concluded that *E*. *coli* strains that possess two or more of these cardinal genes are capable of causing a urinary tract infection.

#### Antibiotic resistance

Acquired antibiotic resistance genes were identified in the assembled genomes using CGE ResFinder 2.1 [14]. The minimum percentage of the gene length detected and the identity threshold were set to 60% for a positive match to be identified between a target genome and the reference database.

## Results

### Genome Sequencing

The Miseq sequencing runs returned an average of 1 597 365 (SD = 486 386) reads per isolate after processing, with an average length of 237 base pairs (bp) (SD = 8.54). The reads from each isolate assembled into an average of 300 contigs (SD = 95.64), with a median coverage ranging from 41.5 to 128.97 between isolates (excluding singletons). Neither the number of reads nor the total number of base pairs sequenced per isolate predicted the sum of their contig lengths (reads: p = 0.915; total bp: p = 0.214). Therefore, the sum of their contig lengths was considered a reasonable estimate of an isolate’s genome size. The estimated genome size of the *E*. *coli* isolates ranged from approximately 4.7 Mbp to 5.4 Mbp (Table 1).

**Table 1:** Source and Phylogenetic information for *E*. *coli* isolates from drinking water.

Isolate ID	Distribution Network^a^	Date	Estimated Genome Size (Mbp)	Phylogenetic Group	MLST	Likely association/ type
E2013	2	14/12/2010	4.7	A	ST-10	Water^b^
E2014	2	14/12/2010	4.68	A	ST-10	Water^b^
E2016	2	14/12/2010	4.68	A	ST-10	Water^b^
E2018	2	14/12/2010	4.7	A	ST-10	Water^b^
E2009	1	22/12/2010	4.75	A	ST-10	Water^b^
E2065	2	16/03/2011	4.73	A	ST-10	Water^b^
E2064	2	16/03/2011	4.77	A	ST-609	
E2029	1	7/02/2011	4.91	B1	ST-191	Water^c^
E2032	1	7/02/2011	4.83	B1	ST-191	Water^c^
E2035	1	7/02/2011	4.84	B1	ST-191	Water^c^
E2004	1	11/11/2010	4.93	B1	ST-201	
E6822	1	3/03/2014	5.11	B1	ST-223	EHEC^d^
E2051	2	1/03/2011	5.17	B1	ST-317	
E2003	1	11/11/2010	4.76	B1	ST-942	
E2074	1	13/04/2011	4.97	B1	ST-2307	
E2048	1	17/03/2011	5.01	B1	ST-2178	EPEC^e^
E2075	1	20/04/2011	4.78	B1/C	ST-1125	
E2059	2	28/02/2011	5.28	B2	ST-95	ExPEC^f^
E2038	Unknown		4.99	B2	ST-372	ExPEC^g^
E6649	3	4/07/2013	5.18	B2	ST-1386	
E2062	2	16/03/2011	4.85	B2	ST-3291	
E2052	2	1/03/2011	5.28	clade I	ST-747	
E2026	1	7/02/2011	4.95	D	ST-69	Human^h^
E4906	3	5/04/2013	5.09	D	ST-394	
E2025	Unknown		5.29	D	ST-3573	
E2039	Unknown		5.03	D	ST-6664	
E2042	Unknown		5.01	D	ST-6664	
E2047	Unknown		5.42	E	ST-6977	

^a^ Samples from unknown distribution networks were provided by the private laboratory.

^b^ Classification was based on full genome sequence similarity to a known bloom strain (see Discussion).

^c^ Classification was based on the possession of few virulence genes (see Tables 2 and 3), detection at multiple points in the distribution network as well as phylogenetic and ST membership (see Discussion).

^d^ Classification was based on the possession of the virulence genes ehxA, stx2A and stx2B (see Table 2), following Robins-Browne et al. [3].

^e^ Classification was based on the possession of the virulence gene eae (see Table 2), following Robins-Browne et al. [3].

^f^ Classification was based on the possession of >2 cardinal ExPEC virulence genes (see Tables 2 and 3) following Johnson et al. [5].

^g^ Classification was based on ST membership (see Discussion) and the possession of a large number of ExPEC virulence genes (see Tables 2 and 3).

^h^ Classification was based on ST membership (see Discussion).

### Phylogenetic Group

**Fig 1:**
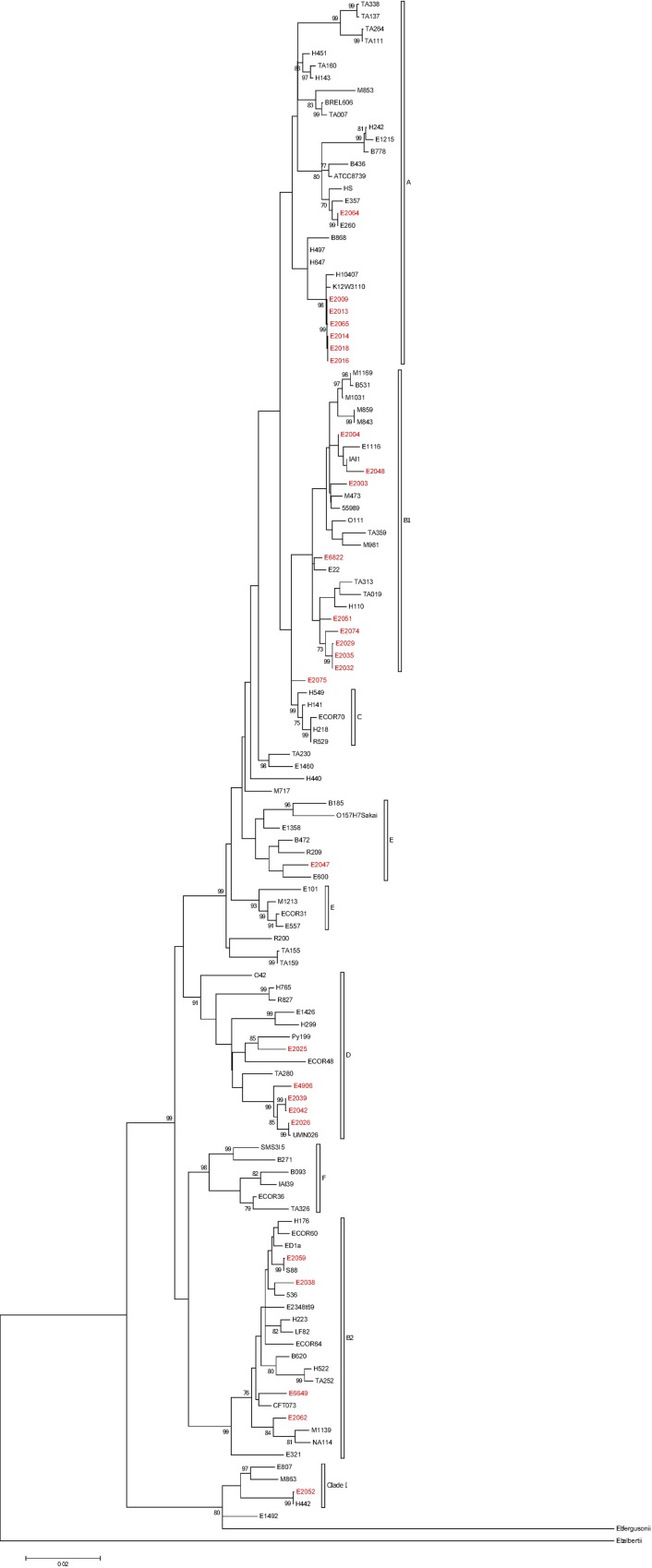
Maximum Likelihood phylogenetic tree of *E*. *coli* isolates based on the General Time Reversible model. Isolates collected from chlorinated drinking water are shown in red. The tree with the highest log likelihood (-31598.3235) is shown. The percentage of trees in which the associated taxa clustered together is shown next to the branches. A discrete Gamma distribution was used to model evolutionary rate differences among sites (6 categories; G = 0.1069) with invariable sites allowed (I = 0.0000% sites). The tree is drawn to scale, with branch lengths measured in the number of substitutions per site.

The maximum likelihood phylogenetic tree revealed that nine of the 28 isolates belonged to phylogenetic group B1, while seven belonged to phylogenetic group A (Table 1 and Fig 1). Five isolated belonged to phylogenetic group D strains and four belonged to phylogenetic group B2. One isolate was found to be a Clade I strain and one isolate was phylogenetic group E. A single isolate (E2075) was found to be intermediate between phylogenetic groups B1 and C (Fig 1).

### MLST

The multilocus sequence types of the isolates are shown in Table 1. Six of the seven group A isolates belonged to ST-10, four of which were isolated from various parts of the same distribution network on the same date (Table 1). In general, the group B1, B2 and D isolates consisted of multiple sequence types that were generally only detected once. However, three B1 isolates with the same sequence type (ST-191) were collected on the same date from various parts of the same distribution network.

### Virulence Associated Genes

Thirty one of the 75 CGE virulence genes (excluding gad) were found in one or more of the isolates, while 22 of the 34 additional extra-intestinal associated virulence genes were detected. Most (43/53) of the virulence genes were detected in fewer than five isolates with 28 genes found in one isolate each (Tables 2 and 3). Two genes (*fimH* and *prfB*) were found in all isolates, while *ompT* (both chromosomal and plasmid) was found in over half the isolates. In all but one case, the matching genome sequences showed an identity of 94.9% or greater to the reference sequences. The majority of matching genome sequences were full length, although, there were 4 cases where only partial sequences were detected in CGE. In isolate E6649, 40 bp was missing from the beginning of the *astA* gene, while, in isolate E2048 there was a 2 bp deletion in the *cif* gene and an 18 bp insertion in the *espJ* gene. In isolate E6822 the partial espP sequence was located at the end of an assembled contig and it is unknown if the isolate contained a full length sequence.

**Table 2:** Summary of virulence associated genes in *E*. *coli* isolates from drinking water.

Isolate ID	number of virulence genes			EPEC gene	EHEC & EPEC gene	EHEC gene	STEC & EHEC genes				EIEC gene	ETEC genes	
	Total	Cardinal ExPEC	Extended ExPEC	bfpA	eae	ehxA	stx1A	stx1B	stx2A	stx2B	ipaH9.8	sta1	ltcA
E2064	2	0	1	-	-	-	-	-	-	-	-	-	-
E2009	3	0	2	-	-	-	-	-	-	-	-	-	-
E2013	3	0	2	-	-	-	-	-	-	-	-	-	-
E2014	3	0	2	-	-	-	-	-	-	-	-	-	-
E2016	3	0	2	-	-	-	-	-	-	-	-	-	-
E2018	3	0	2	-	-	-	-	-	-	-	-	-	-
E2065	3	0	2	-	-	-	-	-	-	-	-	-	-
E2029	4	0	3	-	-	-	-	-	-	-	-	-	-
E2032	4	0	3	-	-	-	-	-	-	-	-	-	-
E2035	4	0	3	-	-	-	-	-	-	-	-	-	-
E2075	4	0	3	-	-	-	-	-	-	-	-	-	-
E2052	5	1	2	-	-	-	-	-	-	-	-	-	-
E2003	5	0	3	-	-	-	-	-	-	-	-	-	-
E4906	6	1	3	-	-	-	-	-	-	-	-	-	-
E2039	6	0	4	-	-	-	-	-	-	-	-	-	-
E2042	6	0	4	-	-	-	-	-	-	-	-	-	-
E2026	7	1	4	-	-	-	-	-	-	-	-	-	-
E2074	7	0	5	-	-	-	-	-	-	-	-	-	-
E2051	8	0	4	-	-	-	-	-	-	-	-	-	-
E2062	8	1	6	-	-	-	-	-	-	-	-	-	-
E2047	9	0	6	-	-	-	-	-	-	-	-	-	-
E2025	9	0	7	-	-	-	-	-	-	-	-	-	-
E2048	11	0	2	-	**+**	-	-	-	-	-	-	-	-
E6649	14	1	10	-	-	-	-	-	-	-	-	-	-
E2004	14	0	11	-	-	-	-	-	-	-	-	-	-
E6822	15	0	7	-	-	**+**	-	-	**+**	**+**	-	-	-
E2059	19	2	15	-	-	-	-	-	-	-	-	-	-
E2038	21	1	15	-	-	-	-	-	-	-	-	-	-

**Table 3:** Virulence associated genes detected^a^ in *E*. *coli* isolates from drinking water.

	Isolate ID	E2064	E2009	E2013	E2014	E2016	E2018	E2065	E2029	E2032	E2035	E2075	E2052	E2003	E4906	E2039	E2042	E2026	E2074	E2051	E2062	E2047	E2025	E2048	E6649	E2004	E6822	E2059	E2038
Cardinal^b^ ExPEC genes	afaD	-	-	-	-	-	-	-	-	-	-	-	-	-	-	-	-	-	-	-	-	-	-	-	-	-	-	-	-
	iutA	-	-	-	-	-	-	-	-	-	-	-	-	-	-	-	-	-	-	-	-	-	-	-	-	-	-	-	-
	kpsE	-	-	-	-	-	-	-	-	-	-	-	+	-	+	-	-	+	-	-	+	-	-	-	+	-	-	+	-
	papC	-	-	-	-	-	-	-	-	-	-	-	-	-	-	-	-	-	-	-	-	-	-	-	-	-	-	+	+
	sfaA	-	-	-	-	-	-	-	-	-	-	-	-	-	-	-	-	-	-	-	-	-	-	-	-	-	-	-	-
Extended ExPEC genes	agn43	-	+	+	+	+	+	+	-	-	-	-	-	-	+	+	+	+	-	-	-	+	+	+	-	-	-	+	-
	cba	-	-	-	-	-	-	-	-	-	-	-	-	-	-	-	-	-	-	-	-	-	-	-	+	-	-	-	-
	clbB	-	-	-	-	-	-	-	-	-	-	-	-	-	-	-	-	-	-	-	-	-	-	-	-	-	-	+	-
	cma	-	-	-	-	-	-	-	-	-	-	-	-	-	-	-	-	-	-	-	-	-	-	-	+	-	-	-	-
	cnf1	-	-	-	-	-	-	-	-	-	-	-	-	-	-	-	-	-	-	-	-	-	-	-	-	-	-	-	+
	ColIa	-	-	-	-	-	-	-	-	-	-	+	-	-	-	-	-	-	-	-	-	-	-	-	-	+	+	-	-
	etsC	-	-	-	-	-	-	-	-	-	-	-	-	-	-	-	-	-	+	-	-	-	+	-	-	+	-	-	-
	fimH	+	+	+	+	+	+	+	+	+	+	+	+	+	+	+	+	+	+	+	+	+	+	+	+	+	+	+	+
	focG	-	-	-	-	-	-	-	-	-	-	-	-	-	-	-	-	-	-	-	-	-	-	-	-	-	-	-	+
	hlyD	-	-	-	-	-	-	-	-	-	-	-	-	-	-	-	-	-	-	-	-	-	-	-	-	-	-	-	+
	hra	-	-	-	-	-	-	-	-	-	-	-	-	-	-	-	-	-	-	+	-	+	+	-	-	-	+	+	+
	ibeA	-	-	-	-	-	-	-	-	-	-	-	-	-	-	-	-	-	-	-	+	-	-	-	+	-	-	-	+
	iha	-	-	-	-	-	-	-	-	-	-	-	-	-	-	-	-	-	-	-	-	-	-	-	-	-	+	-	-
	ireA	-	-	-	-	-	-	-	-	-	-	-	-	-	-	-	-	-	-	-	-	-	-	-	-	-	-	+	-
	iroN	-	-	-	-	-	-	-	-	-	-	-	-	-	-	-	-	-	-	-	-	-	-	-	-	+	-	-	+
	lpfALF82	-	-	-	-	-	-	-	+	+	+	-	-	-	-	-	-	-	-	-	-	+	-	-	+	+	-	-	+
	microcin 47	-	-	-	-	-	-	-	-	-	-	-	-	-	-	-	-	-	-	+	-	-	-	-	-	+	-	-	+
	microcin V	-	-	-	-	-	-	-	-	-	-	-	-	-	-	-	-	-	-	-	-	-	-	-	-	+	-	-	-
	ompT Chromosome	-	-	-	-	-	-	-	-	-	-	+	+	+	-	+	+	+	+	-	+	+	+	-	+	+	+	+	+
	ompT plasmid	-	-	-	-	-	-	-	-	-	-	-	-	-	-	-	-	-	-	-	-	-	-	-	-	-	-	+	-
	papG	-	-	-	-	-	-	-	-	-	-	-	-	-	-	-	-	-	+	-	-	-	+	-	-	+	-	-	-
	senB	-	-	-	-	-	-	-	-	-	-	-	-	-	-	-	-	-	-	-	-	-	-	-	-	-	-	+	-
	sitA	-	-	-	-	-	-	-	+	+	+	-	-	+	-	+	+	+	-	-	+	-	-	-	-	+	-	+	+
	tcpC	-	-	-	-	-	-	-	-	-	-	-	-	-	-	-	-	-	-	-	-	-	-	-	-	-	-	+	-
	tia	-	-	-	-	-	-	-	-	-	-	-	-	-	-	-	-	-	-	-	-	+	+	-	-	-	+	-	+
	traT	-	-	-	-	-	-	-	-	-	-	-	-	-	+	-	-	-	+	+	-	-	-	-	+	+	+	+	-
	upaG	-	-	-	-	-	-	-	-	-	-	-	-	-	-	-	-	-	-	-	-	-	-	-	-	-	-	+	-
	usp	-	-	-	-	-	-	-	-	-	-	-	-	-	-	-	-	-	-	-	+	-	-	-	+	-	-	+	+
	vat	-	-	-	-	-	-	-	-	-	-	-	-	-	-	-	-	-	-	-	+	-	-	-	+	-	-	+	+
	ybtS	-	-	-	-	-	-	-	-	-	-	-	-	-	-	-	-	-	-	-	-	-	-	-	+	-	-	+	+
	astA	-	-	-	-	-	-	-	-	-	-	-	-	-	-	-	-	-	-	-	-	+	+	-	P^c^	-	-	-	-
	celb	-	-	-	-	-	-	-	-	-	-	-	-	-	-	-	-	-	-	-	-	-	-	-	-	-	+	-	-
	cif	-	-	-	-	-	-	-	-	-	-	-	-	-	-	-	-	-	-	-	-	-	-	P^c^	-	-	-	-	-
	espA	-	-	-	-	-	-	-	-	-	-	-	-	-	-	-	-	-	-	-	-	-	-	+	-	-	-	-	-
	espF	-	-	-	-	-	-	-	-	-	-	-	-	-	-	-	-	-	-	-	-	-	-	P^c^	-	-	-	-	-
	espJ	-	-	-	-	-	-	-	-	-	-	-	-	-	-	-	-	-	-	-	-	-	-	+	-	-	-	-	-
	espP	-	-	-	-	-	-	-	-	-	-	-	-	-	-	-	-	-	-	-	-	-	-	-	-	-	P^c^	-	-
	iss	-	-	-	-	-	-	-	-	-	-	-	+	+	+	+	+	+	+	-	-	+	-	-	-	+	+	+	+
	mchC	-	-	-	-	-	-	-	-	-	-	-	-	-	-	-	-	-	-	+	-	-	-	-	-	-	-	-	+
	mchF	-	-	-	-	-	-	-	-	-	-	-	-	-	-	-	-	-	-	+	-	-	-	-	-	+	-	-	+
	mcmA	-	-	-	-	-	-	-	-	-	-	-	-	-	-	-	-	-	-	+	-	-	-	-	-	-	-	-	+
	nleB	-	-	-	-	-	-	-	-	-	-	-	-	-	-	-	-	-	-	-	-	-	-	+	-	-	-	-	-
	pic	-	-	-	-	-	-	-	-	-	-	-	-	-	-	-	-	-	-	-	-	-	-	-	+	-	-	-	-
	prfB	+	+	+	+	+	+	+	+	+	+	+	+	+	+	+	+	+	+	+	+	+	+	+	+	+	+	+	+
	sepA	-	-	-	-	-	-	-	-	-	-	-	-	-	-	-	-	-	-	-	-	-	-	+	-	-	-	-	-
	subA	-	-	-	-	-	-	-	-	-	-	-	-	-	-	-	-	-	-	-	-	-	-	-	-	-	+	-	-
	tir	-	-	-	-	-	-	-	-	-	-	-	-	-	-	-	-	-	-	-	-	-	-	+	-	-	-	-	-

^a^ Genes not detected in any of the drinking water isolates are not shown, with the exception of the cardinal ExPEC virulence genes. For a full list of CGE virulence genes see [13]. Extended ExPEC associated virulence genes not detected in the isolates were: *ColE1*, *iucC*, *microcin B17*, *neuC*, *terC*, *cdiA*, *cloacin*, *cdtB*, and *tsh*.

^b^ *E*. *coli* strains that possess two or more of these cardinal genes may be capable of causing a urinary tract infection, following Johnson et al. [5].

^c^ P = partial length sequence.

**Fig 2:**
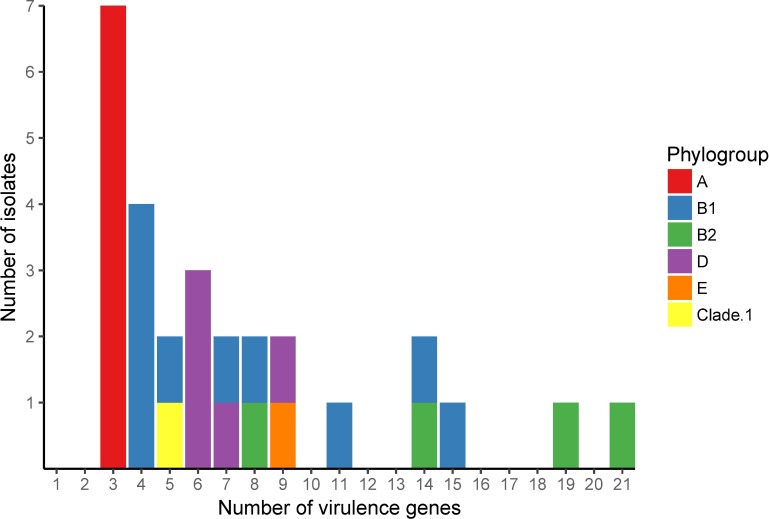
Number of isolates carrying each number of virulence genes. Colours designate the phylogenetic groups of the isolates as indicated in the key.

The number of virulence genes possessed by an isolate ranged from 2 to 21 and varied between the phylogenetic groups (Fig 2). In particular, phylogenetic group A isolates possessed fewer virulence genes than the other phylogenetic groups.

Two isolates could be identified as probable intestinal pathogens based on their virulence gene profiles, following the definition of Robins-Browne et al. [3]. The first isolate, E2048 (group B1) was a putative atypical enteropathogenic (EPEC) strain as it possessed the intimin (*eae*) gene (Table 2). This isolate also carried 10 other virulence genes including genes for a translocated intimin receptor protein, serine protease autotransporter, non-LEE-encoded effector B and type III secretion system. The second isolate, E6822 (group B1), was an enterohemorhagic *E*. *coli* (EHEC) strain as it possessed the enterohemolysin (*ehxA*) gene and two copies of the Shiga toxin 2 subunit genes (Table 2). Isolate E6822 also carried 12 other virulence genes, including genes for the subtilase toxin, increased serum survival, an adherence protein (*iha*) and endonuclease colicin E2.

A further two isolates were identified as putative extra-intestinal pathogens based on their virulence gene profiles. The first such isolate, E2038 (group B2), carried a total of 21 virulence genes, including one cardinal and 15 extended extra-intestinal associated virulence genes. The second isolate, E2059 (group B2), carried nineteen virulence genes, including two cardinal and fifteen extended extra-intestinal virulence associated genes (Tables 2 and 3).

### Antibiotic Resistance

Only one of the 28 isolates was found to possess any of the thirteen acquired antibiotic resistance genes. Isolate E2004 (group B1) was found to possess a full length copy of a tetracycline resistance gene that showed 100% identity to the reference database sequence.

## Discussion

The genetic attributes of an *E*. *coli* isolate can provide valuable insights into its ecological niche and potential for causing disease. In this study we have genetically characterised *E*. *coli* isolates obtained from chlorinated drinking water in South-Eastern Australia. These isolates exhibited a diverse range of genetic profiles. Some of these strains may be of direct concern to human health, while others are likely to be human associated commensals or free-living strains.

The presence of particular genes in an *E*. *coli* strain can give an indication of its ability to cause disease. Only about half of the genes present in a typical *E*. *coli* genome are common to all members of the species [15]. The variable portion of a strain’s genome encodes a range of traits, many of which have been implicated in virulence (for example see [16–18]). In the case of the *E*. *coli* isolated from chlorinated drinking water, most possessed few virulence genes and thus had a limited ability to cause disease. However, four isolates were identified as putative pathogens.

Different types of *E*. *coli* pathogens can be genetically identified as they possess particular combinations of virulence genes [3]. Russo and Johnson [19], defined an *E*. *coli* strain as an putative extra-intestinal pathogen if it possessed currently recognized extra intestinal virulence factors. Additionally, Johnson et al. [5] concluded that *E*. *coli* strains that possess two or more cardinal extra-intestinal virulence genes (*afaD*, *iutA*, *kpsE*, *papC* and *sfaA*) are capable of causing a urinary tract infection. One drinking water isolate (E2059 – group B2) possessed two of these cardinal genes along with 15 other extra-intestinal virulence genes. Therefore, isolate E2059 can be classified as a putative urogenital pathogen. An additional drinking water isolate (E2038– group B2) possessed a very high number of virulence genes, including fifteen extended extra-intestinal associated virulence genes. Furthermore, this isolate was a ST-372 strain, which has previously been implicated in urinary tract infections in both humans and dogs [20]. Thus E2038 is likely to be an extra-intestinal pathogen.

Of the *E*. *coli* strains characterised in this study, two possessed genetic profiles indicative of diarrheal pathogens as defined by Robins-Browne et al. [3]. Isolate, E2048 (group B1) was found to be a putative atypical enteropathogenic (EPEC) strain, while isolate E6822 (group B1) may be an enterohemorhagic *E*. *coli* (EHEC). EPEC strains cause infection by intimately attaching to the gastro-intestinal epithelium. EHEC strains produce toxins called verotoxins that are similar to the Shiga toxin of *S*. *dysenteriae* [21, 22]. Therefore, these strains could present a direct health risk to the public if they were detected at a density where individuals were likely to ingest the Minimum Infective Dose. While the Minimum Infective Dose varies between strains of *E*. *coli*, it is at least one million cells at one time [22]. Thus, as the putative EPEC and EHEC isolates detected in this study each represented a single cell detected in a sole water sample, they were unlikely to be a major health risk at the time of the study.

The different *E*. *coli* phylogenetic groups appear to have different environmental niches and therefore phylogenetic group proportions in a sample collection can give an indication of their primary source. *E*. *coli* is genetically diverse and can be grouped into four main and several minor phylogenetic groups, akin to sub-species [23]. Phylogenetic group B2 strains are more host adapted, showing increased persistence within the gastrointestinal tract but reduced survival in water bodies and soil relative to the other phylogenetic groups [24–27]. By contrast, A and B1 strains survive for longer in the external environmental and only strains from these groups have been identified as free-living in the environment [6]. The different phylogenetic groups also vary in their capacity to cause disease with phylogenetic group B2 strains and to a lesser extent group D strains responsible for the majority of urinary tract infections [4]. A recent study of humans residing in the Australian Capital Territory in South-Eastern Australia during 2011 found that over 38% of the faecal *E*. *coli* isolates belonged to phylogenetic group B2 [28]. By comparison, only approximately 14% of the drinking water isolates belonged to phylogenetic group B2. This suggests that the primary source of the drinking water *E*. *coli* isolates may not have been recent human faecal contamination.

Evidence regarding the possible sources of the *E*. *coli* drinking water isolates also comes from their multi-locus sequence types. Within the phylogenetic groups *E*. *coli* strains can be further divided into different multi-locus sequence types [12]. These sequence types (ST) also vary in their likely source and propensity for causing disease. For instance, ST-69, ST-73, ST-95 and ST-131 have been associated with human extra-intestinal infections [29–31], while most if not all ST-29 strains can cause diarrhoea [32]. Only two of the 28 isolates characterised in this study were human associated STs (E2026 was ST-69 and E2059 was ST-95), further suggesting that many of the isolates were not human derived.

The detection patterns and genetic characteristics of nine (or 32%) of the drinking water isolates were consistent with them being free-living strains. Three phylogenetic B1 isolates with the same sequence type (ST-191) were recovered from the same distribution network on the same day. ST-191 is not a commonly isolated sequence type and is represented by a single non-pathogenic strain in the *E*. *coli* MLST database [20]. Thus, the detection of ST-191 isolates at multiple sites at the same time indicates that they most likely came from the same point of contamination. Four ST-10, phylogenetic groups A, isolates were also recovered from a single distribution network concurrently. These ST-10 strains and an additional two isolates (E2009 and E2065) were virtually identical (based on their full genome sequences, data not shown) to a known phylogenetic group A bloom strain. The detection of a known free-living bloom strain at multiple sites in a distribution network suggests that this strain may have made it through the treatment process.

## Conclusions

The genetic characteristics of the 28 *E*. *coli* isolates sampled from treated drinking water indicate that four isolates were likely human pathogens. However, these isolates were not detected in sufficient numbers to present a risk to public health. An additional isolate was identified as a human associated strain. Nine isolates were water associated free-living strains that were unlikely to pose a health risk. The remaining 14 isolates were probably mammal or water associated strains, although this could not be definitively shown. Together these findings suggest that the primary source of the drinking water isolates may have been the environment. These findings are likely to reflect patterns of *E*. *coli* contamination across water distribution networks that undertake similar environmental management of storage dams and that use comparable water treatment processes to those used by the water authorities involved in this study.
